# Significant relationship between musculoaponeurotic attachment of the abdominal and thigh adductor muscles to the pubis: implications for the diagnosis of groin pain

**DOI:** 10.1007/s12565-023-00750-6

**Published:** 2023-11-20

**Authors:** Suthasinee Tharnmanularp, Satoru Muro, Akimoto Nimura, Takuya Ibara, Keiichi Akita

**Affiliations:** 1 Department of Clinical Anatomy, Graduate School of Medical and Dental Sciences, Tokyo Medical and Dental University (TMDU), 1-5-45 Yushima, Bunkyo-Ku, Tokyo, 113-8510 Japan; 2 Department of Functional Joint Anatomy, Graduate School of Medical and Dental Sciences, Tokyo Medical and Dental University (TMDU), Tokyo, Japan

**Keywords:** Athletic groin pain, External oblique, Adductor longus, Rectus abdominis, Gracilis

## Abstract

Groin pain is prevalent in orthopedic and sports medicine, causing reduced mobility and limiting sports activity. To effectively manage groin pain, understanding the detailed anatomy of supporting muscles is crucial. This study aimed to investigate the musculoaponeurotic attachments on the pubis and the relationship among intramuscular aponeuroses of abdominal and thigh adductor musculatures. Macroscopic analyses were performed in 10 pelvic halves. The bone morphology of the pubis was assessed in two pelvic halves using microcomputed tomography. Histological investigations were conducted in two pelvic halves. The external oblique aponeurosis extended to the adductor longus aponeurosis, forming conjoined aponeurosis, which attached to a small impression distal to the pubic crest. The gracilis aponeurosis merges with the adductor brevis aponeurosis and is attached to the proximal part of the inferior pubic ramus. The rectus abdominis and pyramidalis aponeuroses were attached to the pubic crest and intermingled with the gracilis-adductor brevis aponeurosis, forming bilateral conjoined aponeurosis, which attached to a broad area covering the anteroinferior surface of the pubis. Histologically, these two areas of conjoined aponeuroses were attached to the pubis via the fibrocartilage enthesis. Microcomputed tomography revealed two distinctive bone morphologies, a small impression and an elongated osseous prominence on pubis, corresponded to the two areas of conjoined aponeuroses. This study demonstrated close relationships between the aponeurotic attachment of the external oblique and adductor longus, and between the rectus abdominis, pyramidalis, gracilis, and adductor brevis. The findings of aponeurotic complexes would aid in diagnostic and surgical approaches for athletic groin pain.

## Introduction

Groin pain is a common problem in orthopedic and sports medicine, as evidenced by epidemiological findings indicating that groin-related injuries account for 2–10% of athletic injuries, with a higher prevalence observed among males (de Sa et al. 2016; Ekstrand et al. 2011; Via et al. 2019; Mathieu et al. 2022). Sports requiring sudden changes in direction, including football, rugby, ice hockey, and tennis, can cause repeated stress on the pubic symphysis and supporting muscles, thereby leading to reduced mobility and loss of sports activity (Minnich et al. 2011; Mosler et al. 2018; Orchard 2015; Riff et al. 2019; Saito et al. 2021). Previously, osteitis pubis has been considered the primary cause of athletic groin pain (Batt et al. 1995; Kunduracioglu et al. 2007); however, recent studies on magnetic resonance imaging (MRI) have shown that microtears in musculotendinous attachments, including the rectus abdominis and adductor longus, which correspond to the superior cleft sign location (Murphy et al. 2013), and the short adductor tendons, which correspond to the secondary cleft sign location (Brennan et al. 2005), can also contribute to the athletic groin pain (Murphy et al. 2013; Schilders et al. 2007). To understand the significance of cleft signs and prevention of athletic groin symptoms, a detailed anatomical knowledge of these attachment sites and the relationship between supporting muscles is necessary.

Preceding anatomical studies have proposed that the rectus abdominis connects to the adductor longus via the aponeurotic plate (Becker et al. 2010; Mathieu et al. 2022; Robertson et al. 2009). However, the more recent anatomical concepts have revealed complex and diverse connections among the musculotendinous structures attaching to the pubis, including the gracilis, adductor brevis, adductor longus, rectus abdominis, and pyramidalis (Davis et al. 2012; Norton-old et al. 2013; Schilders et al. 2017). Based on anatomy textbook descriptions, the attachment of these tendons is typically described as every single structure attaching to the area on the anterior surface of the pubic body and pubic symphysis (Snell 2012; Standring 2016), regardless of individual tendon connections and characteristics of their conjoined attachments. Thus, the precise anatomical relationship between these tendons as well as the attachment site on the pubis remain inconclusive (Davis et al. 2012; De Maeseneer et al. 2019; Mathieu et al. 2022; Pesquer et al. 2015; Riff et al. 2019; Schilders et al. 2017), and the bone morphology of the attachment site is not yet understood.

We hypothesized the existence of two distinct areas of musculoaponeurotic attachments corresponding to superior and secondary cleft signs. This study aimed to anatomically investigate the musculoaponeurotic attachments on the anteroinferior part of the pubis and the relationship among the intermuscular aponeuroses based on a comprehensive analysis of aponeuroses and bone morphology. The precise anatomical understanding of the attachment and relationships between these structures would aid in clarifying the pathology of groin symptoms and optimizing clinical approaches.

## Materials and methods

### Cadaveric specimen preparations

Fourteen pelvic halves with groins, including seven right and seven left pelvic halves from seven male Japanese cadavers (mean age at death and standard deviation, 80.86 ± 12.54 years) donated to the Department of Clinical Anatomy were used in this study. The donation document format is consistent with the guidelines of the Act on Body Donation for Medical and Dental Education law of Japan. Donors voluntarily declared to donate their remains for educational purposes before they passed away. This voluntary donor system of cadavers is applied throughout Japan, and our study completely adheres to Japanese laws entitled “The Act on Body Donation for Medical and Dental Education” (Act No. 56 of 1983). All cadaveric specimens were fixed in 8% formalin and preserved in 30% ethanol. Specimens with trauma, degenerative disease, or malformation involving the pelvic region were excluded. The study design was approved by the Institutional Review Board of Tokyo Medical and Dental University (M2018-006). The authors hereby confirm that every effort was made to comply with all local and international ethical guidelines and laws concerning the use of human cadaveric donors in anatomical research.

The lower abdomen, pelvic, and proximal thigh regions were obtained en bloc from the cadavers (Baramee et al. 2020; Muro et al. 2021b; Suriyut et al. 2020). The skin and subcutaneous soft tissues were removed. The specimens were randomly divided into 10, 2, and 2 pelvic halves for dissection, microcomputed tomography (micro-CT), and histological analysis, respectively.

### Macroscopic examination of the musculoaponeurotic structures attaching to the pubis

First, the inguinal ligament, femoral triangle, and relevant structures of the lower abdomen and anterior thigh, including the external oblique, gracilis, adductor longus, pectineus, sartorius, iliotibial tract, iliopsoas, and quadriceps, were observed after removing the skin and subcutaneous soft tissues. The femoral nerve, femoral artery, femoral vein, spermatic cord, and testis were identified and removed. Second, the proximal portion of the external oblique was split and reflected laterally. To identify the intermuscular aponeurosis, the muscular portion of the adductor longus was eliminated. The external oblique and adductor longus aponeuroses were detached en bloc from the attachment site, and the common attachment area was observed. Third, to reveal the rectus abdominis and pyramidalis, the rectus sheath was identified and removed. To reveal the intermuscular aponeuroses, the muscular portions of the rectus abdominis, pyramidalis, as well as, gracilis, adductor brevis, and adductor magnus were removed. The relationship between the rectus abdominis, gracilis, and adductor brevis aponeuroses attaching to the pubis was investigated. Fourth, the suspensory ligament of the penis was dissected. To expose the attachment of the gracilis, adductor brevis, and adductor magnus aponeuroses and observe the relationship on the ischiopubic ramus, the penis was removed inferiorly. Lastly, to demonstrate the common attachment area, the rectus abdominis, gracilis, and adductor brevis aponeuroses were detached en bloc from the attachment site.

In the process of dissection, continuity and non-continuity (separation) between the aponeuroses of different muscles were determined based on the following criteria. Continuity was determined when the dense connective tissues of the aponeurosis were intermingled and could not be bluntly separated during the dissection process. Non-continuity was determined when the dense connective tissues of the aponeurosis were not intermingled and were interspersed with loose connective tissues that could be bluntly separated during the dissection process. Continuity and non-continuity were determined by three observers (S. T., S. M., and A. N.).

The width and length of the common attachments on the pubis were quantified utilizing a non-digital Vernier caliper (PITA15, KANON, Yamanashi, Japan), which provides a precision measurement of up to 0.01 mm. Measurements were acquired twice, at intervals of at least 48 h, by a single observer (S.T).

### Morphological analysis of the pubis using micro-CT analysis and chemical debridement

After macroscopic examination, the bony characteristics of the aponeurotic attachment of the external oblique–adductor longus and that of the rectus abdominis–pyramidalis–gracilis–adductor brevis on the pubis were analyzed in two specimens using micro-CT (inspeXio SMX-100CT; Shimadzu Corp., Kyoto, Japan) with 200-μm resolution and ImageJ (version 1.52; National Institutes of Health, Bethesda, MD, USA). After collecting the three-dimensional (3D) images, to ensure that the 3D-CT image configuration accurately represented the bony surface without dissection artifacts, the soft tissues were chemically removed from the same specimens using a 1% sodium hydroxide solution (Wako Pure Chemical Industries, Osaka, Japan).

### Histological analysis of the pubis

To verify how the external oblique, adductor longus, rectus abdominis, pyramidalis, gracilis, and adductor brevis attached to the pubis, the histological analysis was performed in two specimens. En bloc specimens (size, 6 × 7 cm) were harvested using a diamond band pathology saw (EXAKT 312; EXAKT Advanced Technologies GmbH, Norderstedt, Germany). To obtain the blocks, the superior and inferior borders were made at approximately 1 cm superior and inferior to the pubic symphysis, whereas the medial and lateral borders were made at the mid-sagittal line and at approximately 6 cm lateral to the midline, respectively. The tissue blocks were decalcified in Plank-Rychlo solution (AlCl_3_:6H_2_O, 126.7 g/L; HCl, 85.0 mL/L; HCOOH, 50.0 mL/L) (Fukai et al. 2022; Muro et al. 2023; Norose et al. 2022; Plank and Rychlo 1952) for 4 weeks. A graded ethanol series was used for the dehydration process. The immersion and fixation processes took approximately six times longer than usual. The blocks were embedded in paraffin and serially sectioned in sagittal and axial planes into 5-µm-thick slices at 1-mm intervals. The method employed followed the protocol of wide-range serial sectioning outlined in the previous study (Muro and Akita 2023; Muro et al. 2021a, 2019). The Masson trichrome staining protocol was used to process the staining. A high-quality scanner (GT-X980, EPSON, Nagano, Japan) was used to scan all stained slices; subsequently, specific magnification images were collected using a digital slide scanner (NanoZoomer-SQ C13140, HAMAMATSU, Shizuoka, Japan).

### Statistical analysis

The anatomical relationship of the musculoaponeurotic structures attaching to the pubis in 10 pubic halves was described. The average width and length of common attachments were reported as mean ± standard deviation. Data analysis was carried out utilizing Microsoft® Excel for Mac version 16.76 (license 2019). To ascertain the accuracy of the measurements, all specimens were observed bilaterally. 20 measurements were compared between two assessment days by the same scientist. The intraclass correlation coefficients (ICC) scores were calculated, and the ICC score exceeding 0.75 was consider an excellent agreement. All obtained ICC scores were ≥ 0.96 (range, 0.96–0.99).

## Results

### Relationship between the abdominal and thigh adductor muscles attaching to the pubis

**Fig. 1:**
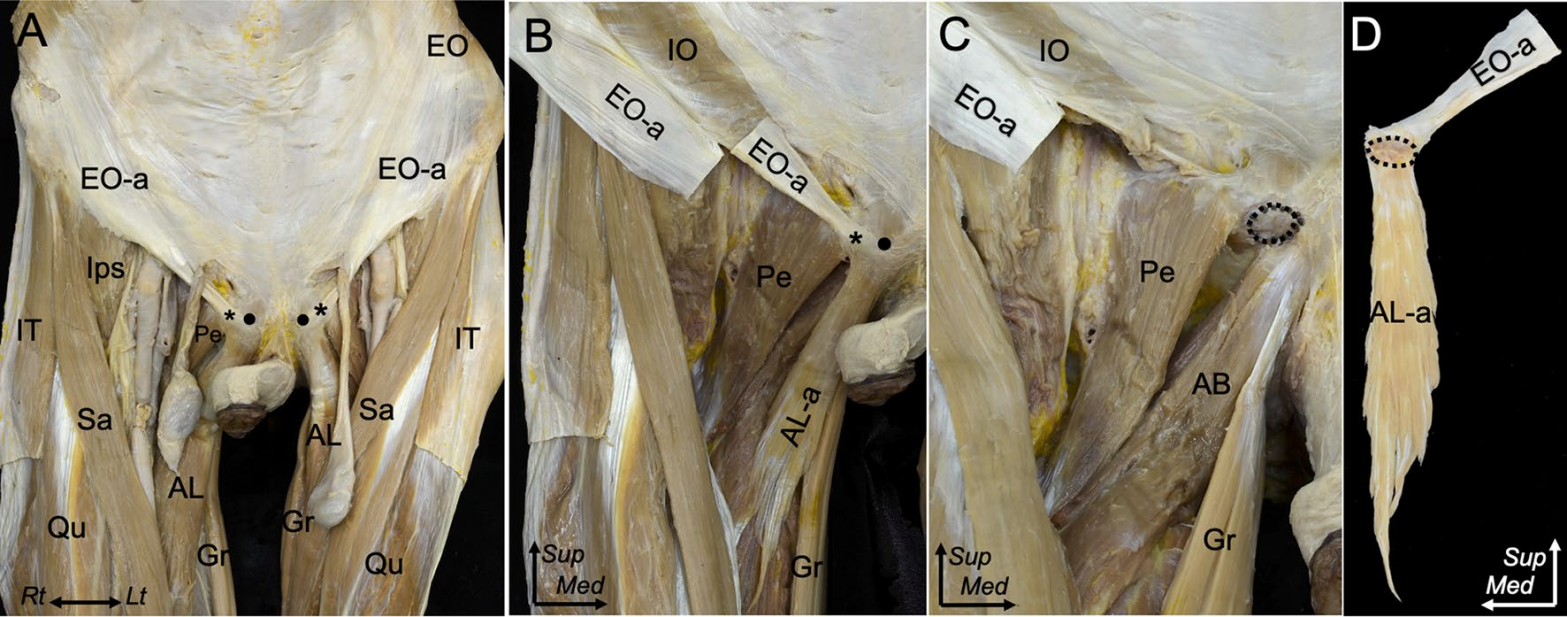
Common aponeurotic attachment of the EO and AL to the pubis.** A** Anterior aspect of the lower abdomen and mid-thigh. The muscles and aponeurosis of the lower abdomen and anterior thigh are identified. **B** Magnified image of **A** on the right side, the femoral nerve, femoral artery, femoral vein, spermatic cord, and testis are removed. The proximal part of the EO-a is split and reflected laterally. The muscular part of the AL is detached. The superficial and distal parts of the EO-a and AL-a are shown to be connected. **C** The EO-a and AL-a are removed from the attachment site. The common attachment of the EO-a and AL-a on the pubis is observed to attach to the small oval impression (black dotted line) distal to the pubic crest (black circle). **D** The posterior aspect of the conjoined EO-a and AL-a is detached from the attachment site. Pubic tubercle (asterisk), pubic crest (black circle)**.** *AB* adductor brevis, *AL* adductor longus, *AL-a* adductor longus aponeurosis, *EO* external oblique, *EO-a* external oblique aponeurosis, *Gr* gracilis, *IO* internal abdominal oblique, *Ips* iliopsoas, *IT* iliotibial tract, *Pe* pectineus, *Qu* quadriceps, *Sa* Sartorius, *Sup* superior, *Med* medial, *Lt* left, *Rt* right

On the anterior aspect of the lower abdomen and mid-thigh, the superficial and distal parts of the external oblique aponeurosis were observed to perpendicularly connect to the adductor longus (Figs. 1A, B). The common attachment of the conjoined external oblique and adductor longus aponeuroses was identified as the small impression distal to the pubic crest (Fig. 1C). The external oblique and adductor longus aponeuroses were observed to be interconnected through the conjoined aponeurosis (Fig. 1D). The connection of external oblique and adductor longus aponeuroses was identified in (10/10) of specimens.

**Fig. 2:**
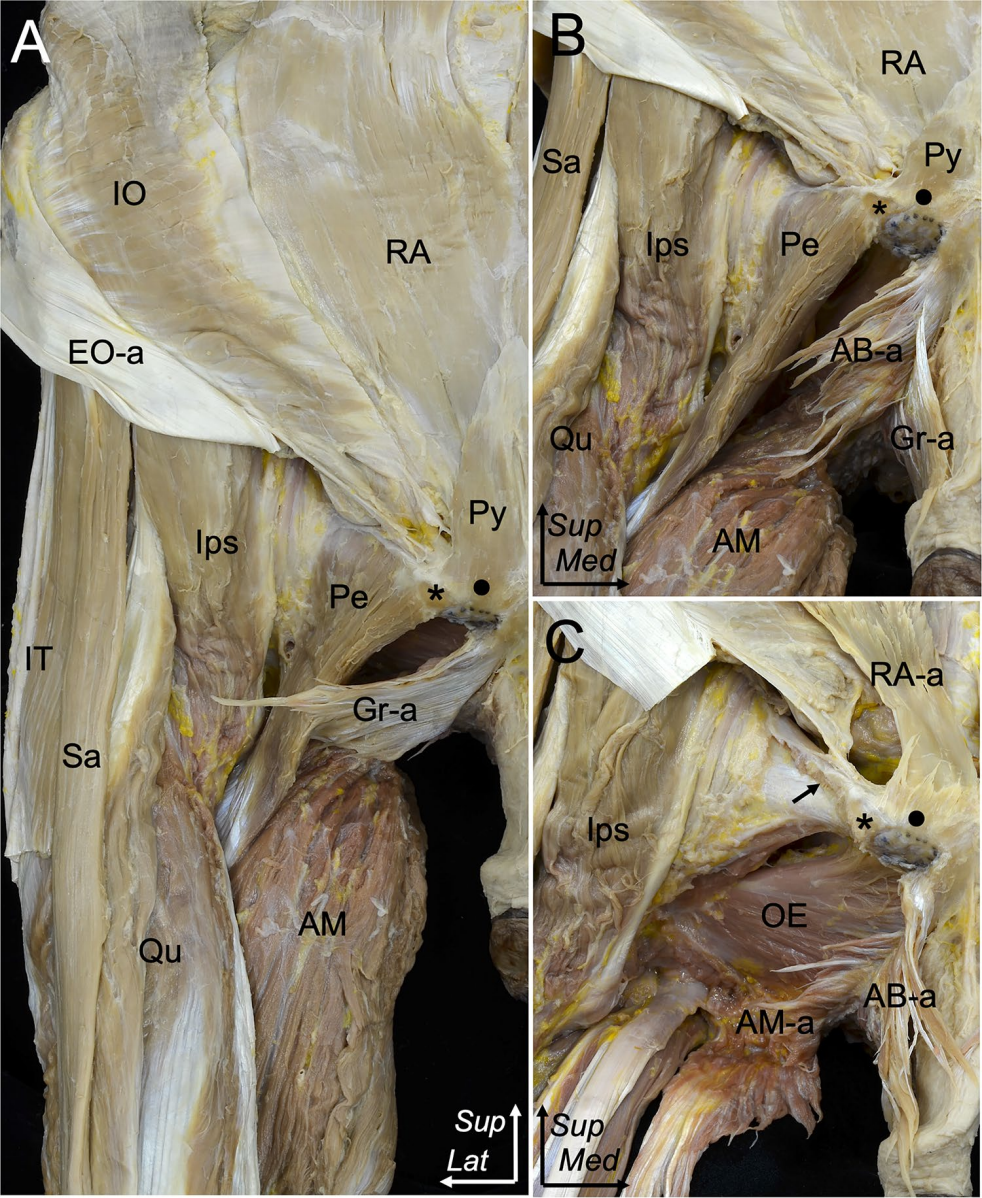
Relationship between the RA-a, Py-a, Gr-a, and AB-a attached to the inferior pubic ramus. **A** Anterior aspect of the abdomen and proximal thigh. On the right site, the EO-a is flipped laterally. The RA and Py are identified. To observe the intermuscular aponeuroses, the muscular parts of the Gr and AB are eliminated. **B** The Gr-a is reflected medially to show the AB-a. The Gr-a is noted to attach superficially to the AB-a. **C** The Gr-a and AB-a are reflected medially. The muscular part of the Pe and AM is removed. Pubic tubercle (asterisk), pubic crest (black circle), pectineal line (black arrow)**.** *AB-a* adductor brevis aponeurosis, *AM* adductor magnus, *AM-a* adductor magnus aponeurosis, *EO-a* external oblique aponeurosis, *Gr-a* gracilis aponeurosis, *IO* internal abdominal oblique, *Ips* iliopsoas, *IT* iliotibial tract, *OE* obturator externus, *Pe* pectineus, *Py* pyramidalis, *Qu* quadriceps, *RA* rectus abdominis, *RA-a* rectus abdominis aponeurosis, *Sa* Sartorius, *Sup* superior, *Med* medial, *Lat*, lateral

**Fig. 3:**
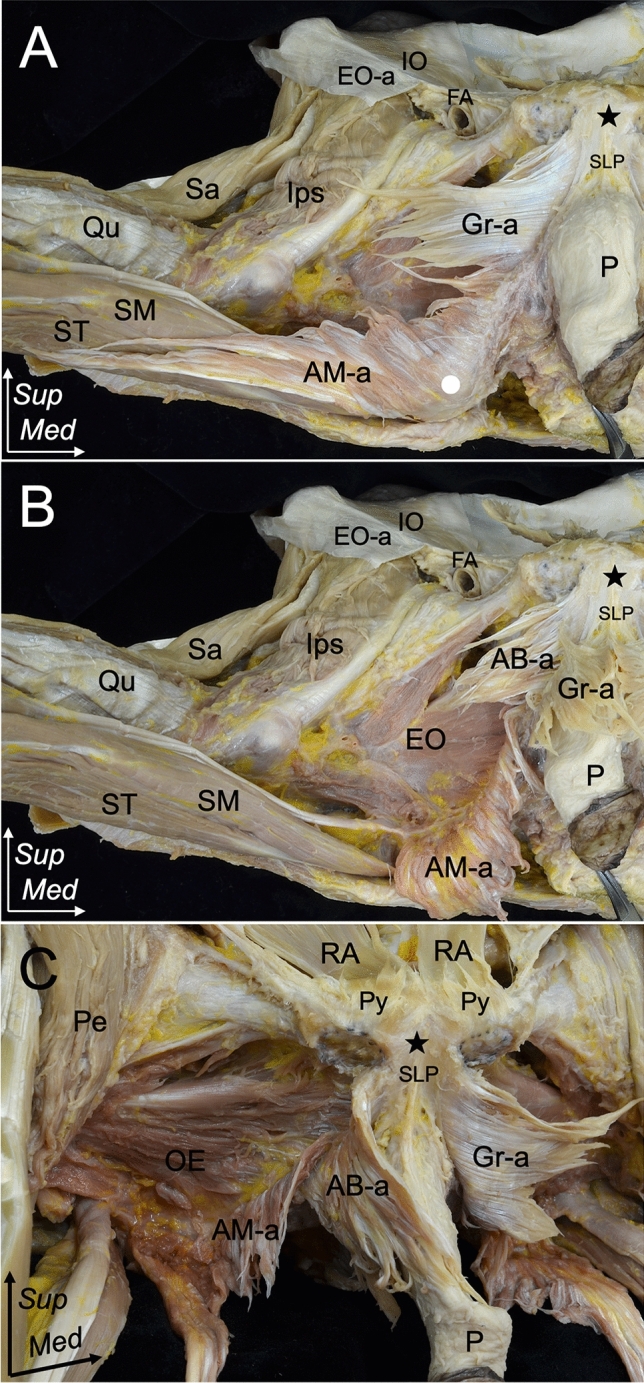
Relationship between the Gr-a, AB-a, and AM-a attached to the inferior pubic ramus. **A**, **B** Inferior aspect of the right pelvic region. The thigh is slightly abducted to demonstrate the relationship between Gr-a, AB-a, and AM-a. **A** The Gr-a together with the AB-a and AM-a are observed to attach along the ischiopubic ramus. The Gr-a and AB-a are attached to the proximal part of the inferior pubic ramus, whereas the AM-a is separately attached to the ischiopubic ramus and ischial tuberosity (white circle). **B** The Gr-a is reflected medially to expose the AB-a. **C** Anterolateral aspect of the pelvic region showing the relationship between the Gr-a along with AB-a and AM-a. The Gr-a and AB-a are reflected medially. The Gr-a is attached superficially to the AB-a and is medially merged to attach to the proximal part of the inferior pubic ramus. Pubic symphysis (black star), pubic tubercle (asterisk). *AB-a* adductor brevis aponeurosis, *AM-a* adductor magnus aponeurosis, *EO-a* external oblique, *FA* femoral artery, *Gr-a* gracilis aponeurosis, *IO* internal oblique, *Ips* iliopsoas, *OE* obturator externus, *P* penis, *Pe* pectineus, *Qu* quadriceps, *Re-a* rectus abdominis aponeurosis, *Sa* Sartorius, *SLP* suspensory ligament of penis, *SM* semimembranosus, *ST* semitendinosus, *Sup* superior, *Med* medial

The gracilis aponeurosis, which was superficial to the adductor brevis aponeurosis, medially merged with the adductor brevis aponeurosis and attached to the proximal part of the inferior pubic ramus (Fig. 2A, B). The pectineus was attached to the pectineal line on the superior pubic ramus via the muscular fiber rather than the aponeurosis (Fig. 2C). Observing from the inferior aspect, the gracilis aponeurosis was located at the proximal half of the ischiopubic ramus, whereas the adductor magnus aponeurosis was located at the distal half (Fig. 3A). The adductor magnus aponeurosis was identified at the deeper layer compared with the gracilis–adductor brevis layer (Fig. 3B). At the anterolateral view, the proximal part of the adductor magnus aponeurosis was separately attached to the distal half of the ischiopubic ramus and ischial tuberosity (Fig. 3C).

**Fig. 4:**
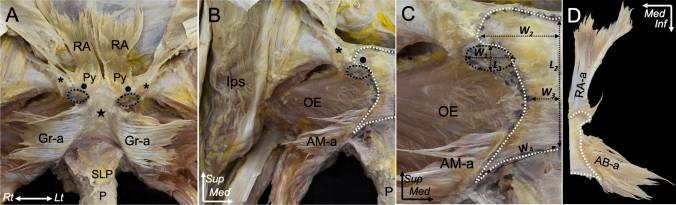
Aponeurotic attachment of the EO, AL, RA, Py, Gr, and AB on the pubis. **A** The suspensory ligament and penis are removed inferiorly. The RA and Py muscular parts are detached. The continuation between the RA-a and Gr-a together with the AB-a on the anteroinferior aspect of the pelvic region is revealed. **B** On the right site, the RA-a, Py-a, Gr-a, and AB-a are observed and removed from the attachment site. The RA-a, along with the Py-a is attached to the pubic crest, continues to the adjoined Gr-a and AB-a, and extends to cover the entire pubic symphysis. The common attachment area of the RA-a, Py-a, Gr-a, and AB-a is marked with the white dotted line, whereas the black dotted line indicates the common attachment area of the EO-a and AL-a. **C** Magnified image of **B**; measurement of the aponeurotic attachment of the EO, AL, RA, Py, Gr, and AB on the anteroinferior aspect of the pubis. The horizontal and vertical diameters of the attachment area are measured using the width (*W1*) and length (*L1*) of the EO and AL common attachments. The width of the superior widest part (*W2*), middle narrowest part (*W3*), and inferior widest part (*W4*) is measured from the midline and the length of the midline (*L2*) is measured for the RA, Gr, and AB common attachments. **D** The posterior aspect of the conjoined RA-a, Py-a, Gr-a, and AB-a is detached from the attachment area. Pubic symphysis (black star), pubic tubercle (asterisk). *AB-a* adductor brevis aponeurosis, *AL-a* adductor longus aponeurosis, *AM-a* adductor magnus aponeurosis, *EO-a* external oblique, *Gr-a* gracilis aponeurosis, *Ips* iliopsoas, *OE* obturator externus, *Py* pyramidalis, *RA-a* rectus abdominis aponeurosis, *SLP* suspensory ligament of penis, *Sup* superior, *Med* medial, *Inf* inferior, *Lt* left, *Rt* right

The pyramidalis was located superficial to the rectus abdominis. The rectus abdominis–pyramidalis aponeurosis was attached not only to the pubic crest but also extended to the gracilis–adductor brevis aponeurosis (Fig. 4A). In addition, the conjoined rectus abdominis–pyramidalis and gracilis–adductor brevis aponeuroses on the right and left sides were fused with each other on the pubic symphysis, creating a connection between the left and right sides. The common attachment was identified as a broad area, extending from the pubic crest to the proximal part of the inferior pubic ramus (Fig. 4B). The width and length of the common attachment area (Fig. 4C) are shown in Table 1. The rectus abdominis, pyramidalis, gracilis, and adductor brevis aponeuroses were observed to be interconnected through the conjoined aponeurosis (Fig. 4D). The connection of rectus abdominis, pyramidalis, gracilis, and adductor brevis aponeuroses was identified in (10/10) of specimen.

**Table 1:** Measurement of the conjoined aponeurotic attachment on the pubis

Location	Mean ± SD (mm)		
EO and AL attachment			
Width (*W*_*1*_)	16.1 ± 4.7		
Length (*L*_*1*_)	13.6 ± 3.0		
RA, Py, Gr, and AB attachment			
Width of the superior widest (*W*_*2*_)	25.0 ± 5.6		
Width of the middle narrowest (*W*_*3*_)	8.8 ± 2.7		
Width of the inferior widest *(W*_*4*_)	27.3 ± 5.4		
Length of the midline (*L*_*2*_)	48.1 ± 3.3		

The area of measurement is demonstrated in Fig. 4C. Descriptive data are presented as mean ± SD

*SD* standard deviation, *EO* external abdominal oblique, *AL* adductor longus, *RA* rectus abdominis, Py pyramidalis, *Gr* gracilis, *AB* adductor brevis

### Pubic morphological characteristics using micro-CT analysis and chemical debridement

**Fig. 5:**
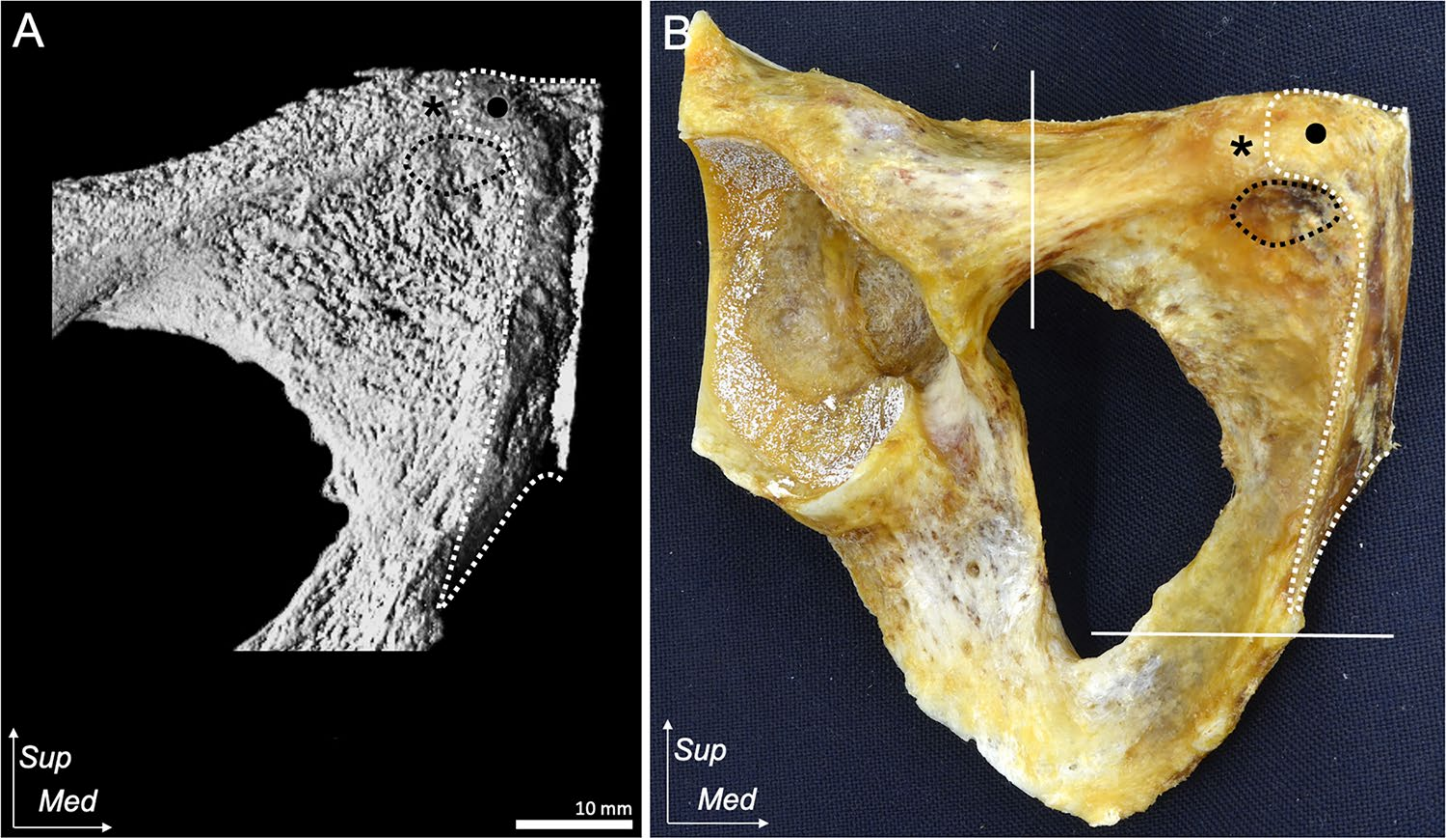
Bone morphology of the aponeurotic attachment site of the EO, AL, RA, Py, Gr, and AB on the pubis.** A** The appearance of the anterior aspect of the right pubis. Micro-CT reveals the osseous morphology of the right pubis. The lateral and inferior borders of A are indicated by white lines in B. The pubic crest (black circle) is located between the pubic tubercle (asterisk) and symphyseal surface. The small oval impression distal to the pubic crest (black dotted line) is observed to correspond to the common aponeurotic attachment of the EO and AL. The broad area (white dotted line), which corresponds to the extended aponeurotic attachment of the RA, Gr, and AB, is identified. **B** To evaluate the correspondence with the micro-CT image, soft tissues are chemically debrided from the same specimen as in **A**. *AB* adductor brevis, *AL* adductor longus, *EO* external oblique, *Py* pyramidalis, *RA* rectus abdominis, *Micro-CT* microcomputed tomography, *Sup* superior, *Med* medial

The pubic crest was identified as a superior border located between the symphyseal surface and pubic tubercle. A small impression was observed distal to the pubic crest, at the medial end of the superior pubic ramus (indicated by the black dotted line in Fig. 5A, B). Medial to the small impression, the elongated osseous prominence was identified extending from the medial end of the superior pubic ramus to the proximal part of the inferior pubic ramus parallel to the symphyseal surface (indicated by the white dotted line in Fig. 5A, B). These bony morphological characteristics can be identified in both the micro-CT image and the actual bone.

### Histological analysis of musculoaponeurotic attachment on the pubis

**Fig. 6:**
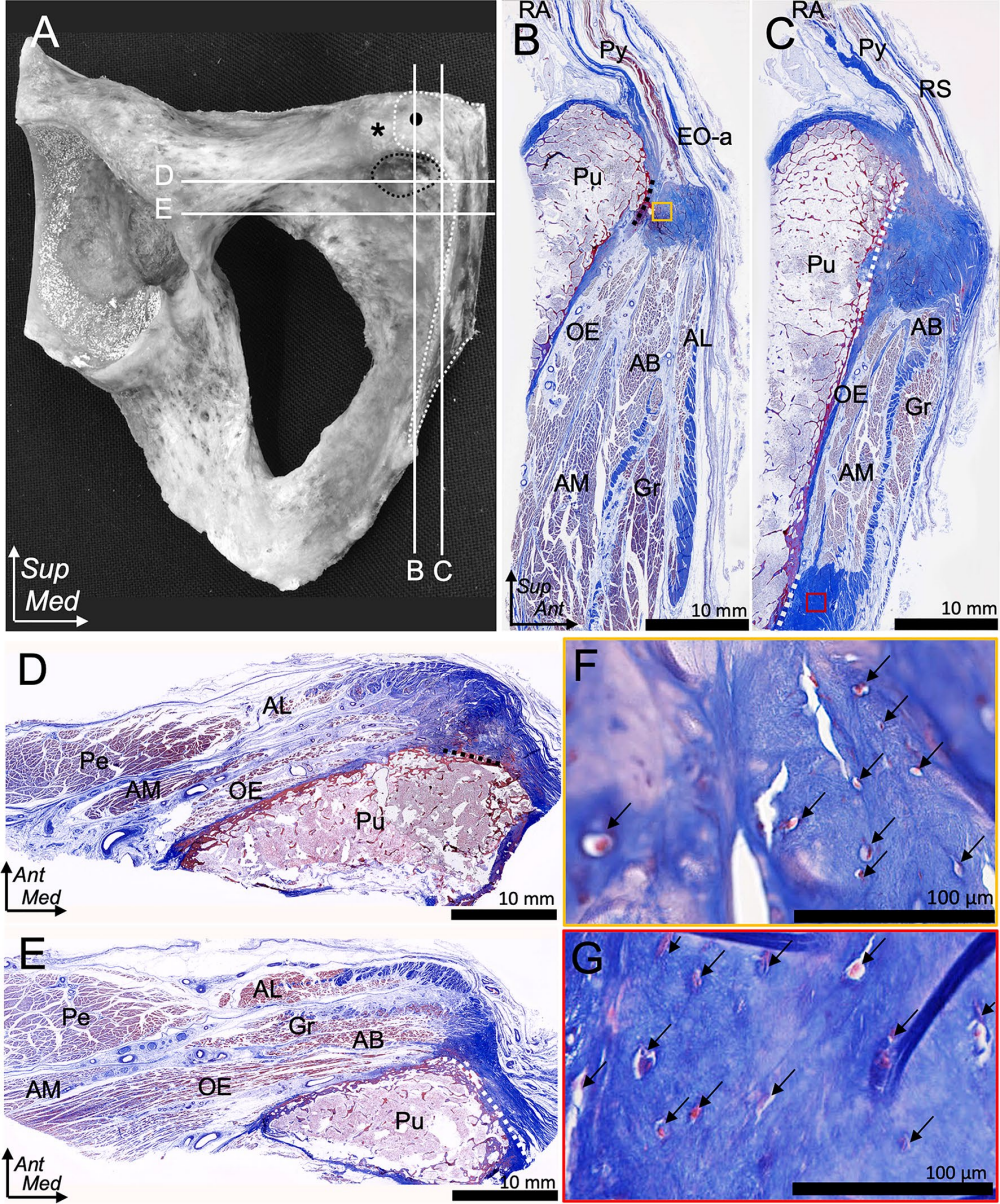
Histological analysis of the tendinous structure attached to the pubis. **A** Image of the anterior aspect of the right pubis with white lines indicating the level of the histological section in **B**–**E**. The pubic crest and pubic tubercle are marked with a black circle and an asterisk, respectively. White and black dotted lines indicate the same attachment area as shown in **B**–**E**. **B** Sagittal section through the pubic crest parallel to the symphyseal surface. The EO-a is observed to continue to the AL through the conjoined aponeurosis, which attaches distally to the pubic crest. The EO-a–AL layer is observed more superficial than the RA–Py–Gr–AB layer. **C** More medial section, 10 mm from the medial surface of B. The RA and Py are observed to connect to the Gr and AB through the common aponeurosis, which attaches to the proximal part of the inferior pubic ramus. The RA–Py–Gr–AB layer is observed more medial and deeper than the EO-a–AL layer. **D** Axial section through the level of the small impression distal to the pubic crest, which corresponds to the EO–AL aponeurosis attachment. **E** More distal, 10 mm from the inferior surface of D, through the Gr–AB aponeurosis attachment. Two separate layers, AL and GR–AB layers, are observed. The AL aponeurosis is identified at the more superficial layer, whereas the GR–AB aponeurosis is observed at the more medial and deeper layer.** F** Magnified image at the yellow square in **B**. **G** Magnified image at the red square in C. The common aponeurotic attachment of the EO–AL and that of the RA–Py–Gr–AB is noted to be fibrocartilaginous enthesis. Chondrocyte cells are indicated by the black arrow.

The sagittal section through the pubic crest parallel to the symphyseal surface showed that the superficial layer of the external oblique was continued to the adductor longus through the conjoined aponeurosis, which attached distally to the pubic crest via the fibrocartilage (Fig. 6A, B). The external oblique–adductor longus aponeurosis was noted at a more superficial layer than the rectus abdominis–pyramidalis–gracilis–adductor brevis aponeurosis layer. In the section 10 mm from the medial side of the section in Fig. 6B, the Re and pyramidalis were connected to the adductor brevis and gracilis through the common aponeurosis and attached to the proximal part of the inferior pubic ramus via the fibrocartilage (Fig. 6A, C). The axial section through the common attachment is presented in Fig. 6D, E. The magnified image of (B) and (C) demonstrated the presence of chondrocyte cells at the fibrocartilaginous enthesis of the external oblique–adductor longus (Fig. 6F) and rectus abdominis–pyramidalis–gracilis–adductor brevis aponeuroses (Fig. 6G), respectively.

## Discussion

**Fig. 7:**
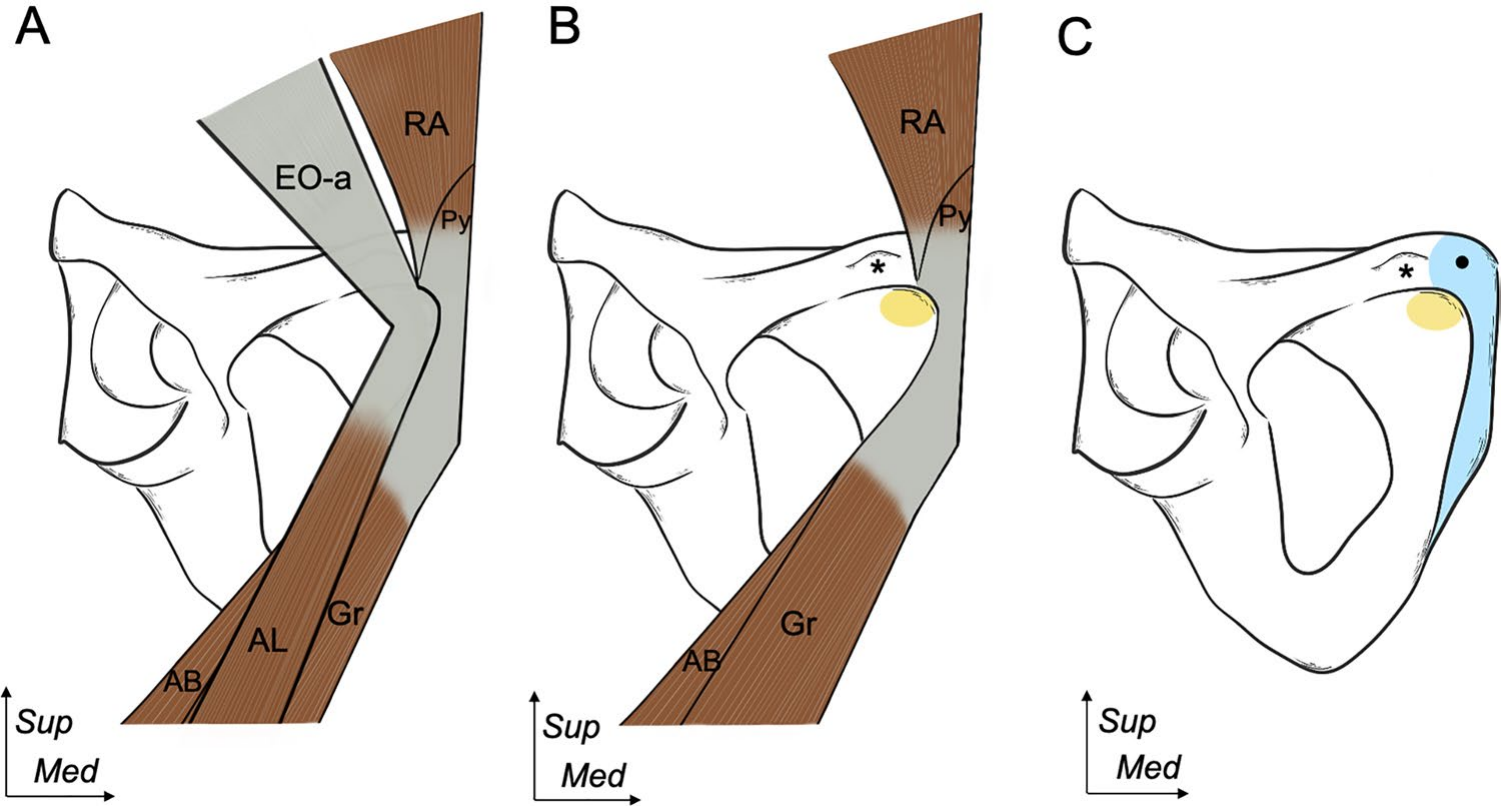
Interrelationships between aponeurotic structures along with the corresponding footprints on the anteroinferior aspect of the pubis.** A** The correlation between the EO-a and AL as well as between the RA, Py, Gr, and AB attaching to the pubis. The EO–AL layer is observed to be more superficial and lateral than the RA–Py–Gr–AB layer. **B** After removing the EO–AL layer. The RA–Py–Gr–AB layer, which is positioned deeper and more medial, is revealed. The yellow area indicates the footprint of the conjoined aponeurosis of the EO and AL. **C** Upon removal of the RA–Py–Gr–AB layer. The blue area highlights the footprint of the conjoined aponeurosis of the RA, Py, Gr, and AB. Pubic symphysis (black star), pubic tubercle (asterisk)**.** *AB* adductor brevis, *AL* adductor longus, *EO* external oblique, *Gr* gracilis, *Py* pyramidalis, *RA* rectus abdominis, *Sup *superior, *Med *medial

The present study showed (1) the connection between the intermuscular aponeurosis of the external oblique and adductor longus, and (2) the connection between the rectus abdominis, pyramidalis, gracilis, and adductor brevis. Two separate areas of musculoaponeurotic attachments were observed on the anteroinferior aspect of the pubis, including the common attachment of the external oblique and adductor longus and that of the rectus abdominis, pyramidalis, gracilis, and adductor brevis (Fig. 7). Furthermore, these common attachments showed the fibrocartilaginous enthesis.

The inconsistency regarding the connection between the adductor longus and rectus abdominis has been debated for centuries, with several studies proposing diverse theories, including the idea that adductor longus was connected to the rectus abdominis via the aponeurotic plate (Becker et al. 2010; Davis et al. 2012; Pesquer et al. 2015; Robertson et al. 2009; Robinson et al. 2007; Zoga et al. 2010). However, diverse conflicting evidence exists in the literature, including the anastomosis between the adductor longus and the contralateral adductor longus and rectus abdominis (Norton-old et al. 2013; Pesquer et al. 2015); the connection between the internal tendon of the gracilis and rectus abdominis (Mathieu et al. 2022; Schilders et al. 2017); and the direct connection between the pyramidalis, anterior pubic ligament, and adductor longus (Schilders et al. 2017). Regarding the attachment of adductor longus and the connection with rectus abdominis, the current study macroscopically and histologically demonstrated the different layers between the external oblique–adductor longus aponeurosis and rectus abdominis–pyramidalis–gracilis–adductor brevis aponeurosis, which corroborated the results of previous studies that refuted the existence of an aponeurotic plate between the adductor longus and rectus abdominis (De Maeseneer et al. 2019; Pieroh et al. 2021). This study employed a range of techniques to conduct a comprehensive investigation into the correlation between each muscle and their adjoining aponeurotic attachments, an aspect that had not been thoroughly explored in previous studies. Conversely, our findings demonstrated that the adductor longus was perpendicularly connected to the distal and superficial parts of the external oblique through the common aponeurosis and affixed to the small oval enthesis distal to the pubic crest.

In addition, previous studies have discussed the fusion between the proximal tendon of the gracilis and adductor brevis (Davis et al. 2012; Pesquer et al. 2015; Pieroh et al. 2021; Robinson et al. 2007). Previous anatomical findings showed that the muscle or tendon component of the adductor brevis appeared to fuse with the proximal tendon of the gracilis (Davis et al. 2012). In contrast, our study observed that only the intermuscular aponeurotic component of the adductor brevis and gracilis formed the conjoined aponeurosis and attached to the proximal part of the inferior pubic ramus. Furthermore, some studies indicated that the lateral tendon of the rectus abdominis was attached to the superior border of the pubis, whereas the internal tendon was attached to the pubic symphysis, connecting to the fascia lata and gracilis (Mathieu et al. 2022; Schilders et al. 2017). However, our findings showed that the rectus abdominis–pyramidalis aponeurosis was attached to the pubic crest and connected to the gracilis–adductor brevis aponeurosis. Moreover, the conjoined rectus abdominis, pyramidalis, gracilis, and adductor brevis aponeurosis on both sides exhibit medial expansion, fusion in the midline, and superficial attachment to the anterior surface of the pubic symphysis, thereby establishing a connection between the left and right sides. Therefore, the current findings oppose the prior reports that suggested the connection between the adductor longus and contralateral adductor longus and rectus abdominis (Norton-old et al. 2013), and the concept of pyramidalis–anterior pubic ligament–adductor longus connection (Schilders et al. 2017).

The fibrocartilage development at the site of tendon and ligament attachments to the bone, known as entheses, is caused by stress concentration (Benjamin et al. 2006; Thomopoulos et al. 2010). Previous studies based on histological (Becker et al. 2010; Davis et al. 2012) and MRI studies (Robinson et al. 2007) have investigated the fibrocartilaginous enthesis of several muscles attaching to the pubic symphysis, including the adductor longus, rectus abdominis, gracilis, and adductor brevis. However, the cross-sectional view was limited to examining only individual fibrocartilaginous enthesis and did not sufficiently demonstrate the connections between each muscle and their common attachments. The current study represented the first comprehensive investigation of the histology of the conjoined aponeurosis attaching to the pubis, using a specialized technique for wide-range serial sectioning (Muro and Akita 2023). The findings clearly demonstrated (1) the connection between the external oblique and adductor longus and (2) the connection between the rectus abdominis, pyramidalis, gracilis, and adductor brevis, which attach to the bone with the help of the fibrocartilage. Based on the concept of the enthesis development (Benjamin et al. 2006; Thomopoulos et al. 2010), we consider that the fibrocartilaginous enthesis of the external oblique–adductor longus and rectus abdominis–pyramidalis–gracilis–adductor brevis resulted from mechanical stress, such as that of repeated stress in different load shifts that may occur during sports activities.

This study emphasizes some clinical implications. First, the superior and secondary cleft signs are diagnostic indicators of injuries to the attachment of different muscle groups to the pubic symphysis, which are widely used in clinical imaging. Specifically, the superior cleft sign indicates an injury to the connection between the rectus abdominis and adductor longus (Murphy et al. 2013; Zoga et al. 2008), whereas the secondary cleft sign indicates an injury to the attachment of the gracilis and adductor brevis (Brennan et al. 2005). These signs are characterized by linear signal hyperintensity that runs parallel to the inferior margin of either the superior or inferior pubic ramus, resulting from localized microtearing (Byrne et al. 2017; De Maeseneer et al. 2019; Zoga et al. 2010). However, based on the dissection and histological analysis of the current study, the adductor longus aponeurosis was perpendicularly connected to the external oblique aponeurosis instead of the rectus abdominis. According to the common attachment site of the external oblique–adductor longus aponeurosis, we considered that the external oblique–adductor longus enthesis could correspond to the superior cleft sign location rather than the adductor longus–rectus abdominis, as described in previous reports. Regarding the secondary cleft sign location, our study verified that the gracilis aponeurosis medially merged to the adductor brevis aponeurosis and attached to the proximal part of the inferior pubic ramus, where it corresponded to the secondary cleft sign location, as reported in previous studies (Byrne et al. 2017; De Maeseneer et al. 2019; Zoga et al. 2010). These findings could aid in diagnosing and understanding the pathology of groin symptoms, including superior and secondary cleft signs.

Our study had some limitations. First, our investigation was restricted to anatomical observations and conducted solely on uninjured specimens. Therefore, we are unable to provide definitive proof regarding the pathomechanism of the observed cleft signs. Second, as we could only include old-age cadavers, the age of the cadavers used in our study did not match that of previous clinical studies (Brennan et al. 2005; Byrne et al. 2017; Cunningham et al. 2007; O'Connell et al. 2002; Saito et al. 2021; Zoga et al. 2008). Lastly, the number of specimens employed for the histological analysis was limited. Further research through additional biomechanical studies or clinical case imaging is required to confirm the validity of our findings.

## Conclusion

The findings of this study indicate a significant correlation between the musculoaponeurotic attachment of abdominal and thigh adductor muscles to the pubis. This study further unveiled two distinct areas of musculoaponeurotic attachments on the anteroinferior aspect of the pubis, including the common attachment of the external oblique and adductor longus and that of the rectus abdominis, pyramidalis, gracilis, and adductor brevis. Examining aponeurotic complexes could potentially provide valuable insights into diagnostic and surgical approaches for individuals experiencing athletic groin pain.

## Data Availability

The data that support the findings of this study are available from the corresponding author upon reasonable request.

## Abbreviations

AB: Adductor brevis
AL: Adductor longus
EO: External oblique
Gr: Gracilis
Py: Pyramidalis
RA: Rectus abdominis
